# Placing Greater Torque at Shorter or Longer Muscle Lengths? Effects of Cable vs. Barbell Preacher Curl Training on Muscular Strength and Hypertrophy in Young Adults

**DOI:** 10.3390/ijerph17165859

**Published:** 2020-08-13

**Authors:** João Pedro Nunes, Jeferson L. Jacinto, Alex S. Ribeiro, Jerry L. Mayhew, Masatoshi Nakamura, Danila M. G. Capel, Leidiane R. Santos, Leandro Santos, Edilson S. Cyrino, Andreo F. Aguiar

**Affiliations:** 1 Metabolism, Nutrition, and Exercise Laboratory, Physical Education and Sport Center, Londrina State University, Londrina 86057-970, PR, Brazil; alex.sribeiro@kroton.com.br (A.S.R.); leandro.santos.sm@gmail.com (L.S.); edilsoncyrino@gmail.com (E.S.C.); 2 Center for Research in Health Sciences, University of Northern Paraná, Londrina 86041-140, PR, Brazil; jeferson1995lucas@gmail.com (J.L.J.); danilamcapel@gmail.com (D.M.G.C.); leydremigio@gmail.com (L.R.S.); afaguiarunesp@gmail.com (A.F.A.); 3 Exercise Science Program, Truman State University, Kirksville, MO 63501, USA; jmayhew@truman.edu; 4 Niigata University of Health and Welfare, Niigata 950-3198, Japan; masatoshi-nakamura@nuhw.ac.jp

**Keywords:** variable resistance, muscle architecture, exercise selection, strength training, Scott curl

## Abstract

Muscular strength and hypertrophy following resistance training may be obtained in different degrees depending on the approach performed. This study was designed to compare the responses of the biceps brachii to two preacher curl exercises, one performed on a cable-pulley system (CAB; in which a greater torque was applied during the exercise when elbows were flexed and biceps shortened) and one performed with a barbell (BAR; in which greater torque was applied when the elbows were extended and biceps stretched). Thirty-five young adults (CAB: 13 men, 5 women; BAR: 12 men, 5 women; age = 24 ± 5 years) performed a resistance training program three times per week for 10 weeks, with preacher curl exercises performed in three sets of 8–12 repetitions. Outcomes measured included elbow flexion peak isokinetic torque at angles of 20°, 60°, and 100° (considering 0° as elbow extended), and biceps brachii thickness (B-mode ultrasound). Following the training period, there were significant increases for both groups in elbow flexion peak torque at the 20° (CAB: 30%; BAR = 39%; *p* = 0.046), 60° (CAB: 27%; BAR = 32%; *p* = 0.874), and 100° (CAB: 17%; BAR = 19%; *p* = 0.728), and biceps brachii thickness (CAB: 7%; BAR = 8%; *p* = 0.346). In conclusion, gains in muscular strength were greater for BAR only at longer muscle length, whereas hypertrophy was similar regardless of whether torque emphasis was carried out in the final (CAB) or initial (BAR) degrees of the range of motion of the preacher curl in young adults.

## 1. Introduction

Depending on the purpose of the resistance training program, specific adjustments in the training schedule should be made [1]. Whilst the effects of some variables, such as training volume and intensity, have been widely investigated, exercise selection to elicit specific effects on muscle has not [2]. It is worth noting that exercise selection in resistance-training programs is generally based on acute biomechanical studies [2,3]. However, given the gap between acute and chronic responses, long-term investigations are needed to determine the effects of executing different exercises on primary outcomes of strength and hypertrophy.

For muscular strength, responses are directly dependent on the task practiced [4,5]. That is, regardless of the specific training variable manipulated, more significant performance increases tend to be observed when the strength tests are similar to the exercise task and intensity of load employed during training [5,6,7,8]. For example, the benefit of traditional linear periodization programs for improving one-repetition maximum strength seems to be because participants train with higher loads during sessions near post-training evaluation [4]. Moreover, strength adaptations seem to be vector and angle specific, such that greater results tend to be observed in the direction and at the specific angle where higher torque was applied during the execution of the exercise [6,8,9]. For muscle hypertrophy, it seems that the muscle portions that show greater acute activation tend to have greater long-term growth compared to other portions [10,11,12].

In the same way, a recent review with meta-analysis regarding isometric training indicated that training at longer muscle lengths was superior to training at shorter lengths for improving strength throughout a wide range of motion [13]. Furthermore, it was observed that greater strength gains were obtained in the position trained compared with other tested angles [13], following the specificity principle [14,15]. Moreover, training at longer muscle lengths tended to produce greater hypertrophy [13]. This seems to most likely occur by altering joint moment arm, providing greater mechanical tension [13,16], which has been shown to be important for muscle growth [1]. Given that muscle tends to grow where it experiences the highest levels of tension, and more sarcomerogenesis may occur to adapt the muscle to receive high torques when stretched [16,17], greater hypertrophy in training with elongated muscle would be plausible.

**Figure 1:**
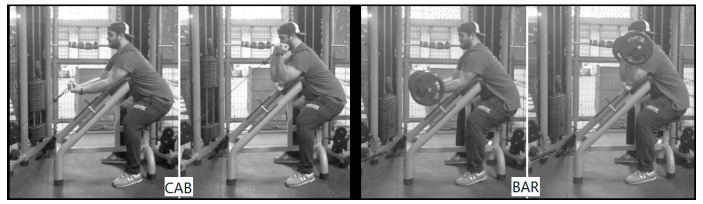
Examples of how cable (CAB) and barbell (BAR) preacher curls were performed.

It remains unclear whether strength and hypertrophy changes occur in dynamic exercises when the highest torque application is during different positions of the range of motion. That is to say, will larger strength and hypertrophy gains occur when the highest torque is produced in a specific range of motion? Therefore, this study was designed to compare the responses to two preacher curl exercises, in which maximal force was applied in a shortened or an elongated position following 10 weeks of progressive resistance training in young adults. In order to investigate these points, participants were invited to perform the exercises on a cable-pulley system (CAB), in which a greater torque was applied during the exercise when elbows were flexed and biceps shortened, or with a barbell (BAR), in which greater torque was applied when the elbows were extended and biceps elongated (Figure 1). It was hypothesized that the increase in muscular strength would be angle-specific [13,15], and greater hypertrophy would be observed for the BAR group [13].

## 2. Materials and Methods

### 2.1. Experimental Design

This study is part of a large research project designed to analyze the effects of whole-body resistance-training protocols in untrained young adults, where participants performed eight exercises, in the following sequence: bench press, leg-press, wide-grip lat-pulldown, leg extension, preacher curl (cable or barbell), leg curl, triceps pushdown, and shoulder lateral raise. The current investigation was executed over a period of 14 weeks, in which weeks 1–2 were used for familiarization with the exercises, week 3 and week 14 were used for pre- and post-training strength and hypertrophy measurements, respectively, and the training program was carried out for 10 weeks (weeks 4–13). During weeks 1–2, participants performed the preacher curl with both the cable and barbell apparatus, alternating between sessions. For the 10-week specific training period, participants were randomly divided into two groups for the CAB or BAR training. Maximum arm flexion strength was assessed on an isokinetic dynamometer, while hypertrophy was analyzed by changes in biceps brachii thickness. Written informed consent was obtained from all participants after a detailed description of study procedures was provided. This investigation was conducted according to the Declaration of Helsinki and was approved by the University Ethics Committee (number: 01993418.9.0000.0108).

### 2.2. Participants

Recruitment was carried out through social media and home delivery of flyers in the university area. Interested participants completed detailed health history and physical activity questionnaires and were subsequently admitted if they met following inclusion criteria: 18–35 years old, free from cardiac, orthopedic, or musculoskeletal disorders that could impede exercise practice, did not consume drug or supplement ergogenic aids, and not involved in the practice of resistance training over the 6 months before the start of the study. From the 112 volunteers, 74 met the criteria, but only 57 remained after the familiarization period and were evaluated at baseline, and initiated the training sessions. During the training period, participants who obtained six absences from training sessions (resulting in an attendance < 80% of the total number of sessions) were withdrawn from the training program. Thirty-five participants (CAB: 13 men, 5 women; BAR: 12 men, 5 women) ultimately completed the study and were included for final analyses (age = 23.7 ± 5.3 years; body mass = 71.7 ± 12.2 kg; stature = 172.9 ± 8.6 cm; body mass index = 25.0 ± 3.6 kg/m²). This final sample size is considered satisfactory (*n* > 16 per group) to achieve a power of 0.8 and an α of 0.05 for improving muscle morphology with an effect size of 0.50 [18].

### 2.3. Muscular Strength

Elbow flexion strength was determined from the concentric peak torque (Nm) of the dominant arm, assessed on an isokinetic dynamometer (Biodex Medical Systems Inc., System 3 model, Shirley, MA, USA). Upon arriving at the laboratory, participants were positioned in the sitting position in an 85° hip flexion, according to anatomical position. The axis of the dynamometer lever was aligned with the lateral epicondyle of the humerus. The elbow was supported on a padded shelf with the shoulder flexed at an angle of 60°, similar to the preacher bench. Two straps were secured to keep the torso stabilized. Gravity correction was applied at 0° (parallel to the horizon position), and cushioning was set at moderate, according to the manufacturer’s recommendations. During the test, participants were instructed to hold the lever firmly with the hand in a supine position and were admonished to pull it as strongly and quickly as possible. Each participant performed 10 attempts of elbow flexions through a range of motion of 0–120° at an angular velocity of 60°/s, with 3–5 s rest between them. Maximum torques at 20°, 60°, 100° were recorded. Although the test standardized by the equipment was for elbow flexion/extension, participants completed only elbow flexion with maximum force and returned to the starting position (i.e., elbow extensions) by relaxing the limb. Test-retest (separated by 72 h) indicated an intraclass correlation coefficient of 0.95, 0.96, and 0.89, and a standard error of measurement of 3.8 Nm, 3.2 Nm, and 3.1 Nm, for maximum torques at 20°, 60°, and 100°, respectively [19].

### 2.4. Muscle Thickness

Measures of biceps brachii thickness were obtained using a B-mode ultrasound with a 10.0-MHz linear probe (Esaote, MyLabTM30 model, Florence, Italy) by the same experimenter, blinded to group allocation. Upon arrival at the laboratory on measurement days, participants had to verbally certify that they had been fasting for 8 h and had not performed vigorous exercise for the previous 48 h. Ultrasound measurements started after participants were lying down in prone position for 10 min. Images were acquired halfway the distance between the acromion process of the scapula and the olecranon process of the ulna. Water-soluble transmission gel was applied over the skin of the muscle being assessed with caution not to depress the muscle tissue. Images were acquired with the probe placed perpendicular to the tissue interface and were recorded at 25 Hz, with a field of view of 60 mm depth. Two experimenters participated in measurement procedures so that one handled the probe, and the other was responsible for freezing the images (once the first considered that image quality was satisfactory). An image as an example of how biceps muscle thickness was measured can be seen in the Supplementary Material. The muscle thickness was defined as the distance between the superficial and deep aponeuroses.

### 2.5. Preacher Curl Training

The supervised resistance-training program was performed three times per week (Mondays, Wednesdays, and Fridays) in the afternoon period for 10 weeks. This training length has previously been shown to provide adequate time for hypertrophy to occur [18,20,21]. The preacher curl exercises (Ipiranga, Fitness Line, Presidente Prudente, Brazil) were performed in 3 sets of 8–12 repetitions, in the maximum range of motion, in a tempo of 1:2 s (concentric and eccentric phases, respectively). When near to momentary muscular failure (last ~2 repetitions), participants were released to carry out the movement at a capable velocity. The rest between sets was 90–120 s. Training loads were initially selected based on the training logs of the familiarization sessions and were fine-adjusted following a protocol previously described [22,23] so that the participants used a load related to 8–12 RM. Loads were progressively increased each week by ~5%, as recommended [24], according to the number of repetitions performed during training sessions to ensure that the participants kept performing the sets to (or very near to) failure in the established repetition zone [25]. For both groups, participants were instructed to hold the straight handle (or the bar) with hands supinated and shoulder-width apart. Figure 1 illustrates how exercises were performed.

### 2.6. Statistical Analyses

Normality and homogeneity of variances were checked by the Shapiro–Wilk and Levene’s tests, respectively. Non-normal variables (peak torque at 20° and 60°) were analyzed with log_10_ adjustment. Training effects were examined with analysis of covariance of the raw difference between pre- to postintervention measures, with baseline values as a covariate to eliminate any possible influence of initial score variances on outcomes. Interpretation of data was based on 95% confidence intervals (CI) of the change score (e.g., when the Bonferroni-adjusted 95% CI of the raw delta did not overlap 0, there was a difference between baseline score). The *p* values of the analysis of covariance for group comparisons were also presented. Additionally, three-way repeated-measures analysis of variance, comparing times (pre vs. post), groups (CAB vs. BAR), and sexes (men vs. women), was performed to determine whether responses to CAB vs. BAR training interacted with sex. A *p* < 0.05 was accepted as statistically significant. Effect size (ES) was calculated as post-training mean minus pretraining mean, divided by pooled pretraining standard deviation [26]. An ES of 0.00–0.19 was considered as trivial, 0.20–0.49 as small, 0.50–0.79 as moderate, and ≥0.80 as large [26]. The data were expressed as mean, standard deviation, and 95% CI. The data were stored and analyzed using JASP software (Jasp Stats, v.1.0. Amsterdam, The Netherlands).

## 3. Results

No significant time × group × sex interaction was observed for elbow flexion peak torque at 20° (*p* = 0.241; ES of the change: women = 0.78; men = 0.76), at 60° (*p* = 0.286; ES of the change: women = 0.56; men = 0.85), and at 100° (*p* = 0.888; ES of the change: women = 0.42; men = 0.59), nor for biceps muscle thickness (*p* = 0.382; ES of the change: women = 0.35; men = 0.42), indicating that responses to cable or barbell preacher curls were similar between men and women.

**Figure 2:**
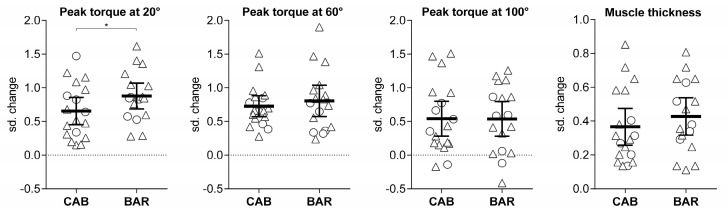
Individual standardized (sd.) changes (post-training minus pretraining value, divided by pooled pretraining standard deviation) for elbow flexion isokinetic peak torque at 20°, 60°, and 100°, and muscle thickness according to groups that performed cable (CAB, *n* = 18) or barbell (BAR, *n* = 17) preacher curl exercises. The horizontal lines represent mean and 95% confidence intervals. Triangles represent men, and circles represent women. * *p* < 0.05 between groups.

For elbow flexion peak torque at 20°, significant increases were observed for both CAB (pre = 30 ± 13 Nm, post = 38 ± 12 Nm; ES = 0.65; +30%) and BAR (pre = 31 ± 14 Nm, post = 42 ± 14 Nm; ES = 0.86; +39%), with greater gains for the BAR group (*p* = 0.046). For elbow flexion peak torque at 60°, significant increases were observed for both CAB (pre = 32 ± 12 Nm, post = 40 ± 12 Nm; ES = 0.73; +27%) and BAR (pre = 30 ± 12 Nm, post = 39 ± 14 Nm; ES = 0.79; +32%), without significant difference between them (*p* = 0.874). For elbow flexion peak torque at 100°, significant increases were observed for both CAB (pre = 31 ± 11 Nm, post = 36 ± 11 Nm; ES = 0.54; +17%) and BAR (pre = 26 ± 9 Nm, post = 32 ± 8 Nm; ES = 0.52; +20%), without significant difference between them (*p* = 0.728). For biceps brachii thickness, significant increases were observed for both CAB (pre = 25 ± 5 mm, post = 27 ± 6 mm; ES = 0.37; +7%) and BAR (pre = 24 ± 4 mm, post = 26 ± 4 mm; ES = 0.35; +8%), without significant difference between them (*p* = 0.346). Individual standardized changes according to groups are displayed in Figure 2.

## 4. Discussion

The main finding of the present study was that biceps brachii muscle adaptations following a 10-week training program were almost identical regardless of whether peak torque emphasis was carried out in the final degrees (CAB) or initial degrees (BAR) of the range of motion in young adults. Our hypothesis that greater strength and hypertrophy would occur when peak toque was generated early in the range of motion was not confirmed. A significant advantage, albeit of small magnitude (ES = 0.23), was observed for BAR on improving extended elbow strength (at 20°) compared to CAB, which was the portion of the range of motion in which the BAR condition had the greatest torque during exercise, indicating a specificity of the adaptation. However, to comprehensively confirm that strength gains were indeed angle specific, the CAB group should have had a larger increase in peak torque at 100°, which was not the case.

Given that performing isometric training at longer muscle lengths may improve strength to adjacent angles of the trained one [13,14], it can be supposed that BAR induced greater strength gains at 20°, leading to a significant difference compared to CAB, but also prompted gains to the other tested angles. That is, the gains induced by BAR at the angle where CAB would present an advantage (i.e., at 100°) were sufficient to be similar to those obtained by CAB. On the other hand, since training at shorter muscle lengths may give gains that are more angle specific and may not result in significant gains beyond the trained angle [13,14], this propitiated that there was a significant difference between groups in the angle distant (i.e., 20°) to those the CAB trained with greater torque (i.e., ~100°). These findings are very similar to those presented by Thépaut-Mathieu et al. [14] with isometric training. Young male adults trained isometric elbow flexions at 25°, 80°, or 120° for 5 weeks, 3x/week. Strength gains for the 25° group were higher at 25° and similar at 80° compared to the 80°-group. For the 80°-group, responses were greater at 80° and similar at 120° compared to the 120°-group. Similar responses have also been observed in other joints [13].

After the training period, the CAB and BAR groups increased biceps brachii thickness in a similar magnitude, i.e., regardless of the muscle length with which the greater peak of torque was imposed to biceps in the preacher curl. The hypothesis that greater hypertrophy would be observed for the BAR group was not confirmed. The rationale was based on the greater internal physiological stress which would be produced by the muscle at a longer muscle length compared to shorter muscle lengths, due to the difference in moment arm length between conditions. At longer muscle lengths, there are more interactions between actin and myosin filaments, thus, as a result of the increased mechanical stress placed on the elongated muscle, greater hypertrophy would occur [13,16,27,28], which was not the case herein. However, on the other hand, greater metabolic stress may be obtained when focusing the exercise execution training with short lengths [29]. Thus, the balance between lower and greater torque at the distinct phases of the movement (i.e., lower torque for CAB and greater for BAR at the initial angles; and vice-versa) and between mechanical and metabolic stress—which are factors, among others, important for muscle growth [1]—may explain the similarity of results between CAB and BAR.

Previous findings also on biceps brachii, from Pinto et al. [20], showed similar hypertrophy following preacher curl at full (0–130°) and partial (50–100°) ranges of motion. Conversely, studies on the quadriceps muscle indicated hypertrophic benefits to training at longer muscle lengths [27,30]. After lower-limb training at longer (40–90° of knee flexion; 0° = full knee extension) and shorter (0–50°) muscle lengths, McMahon et al. [30] observed a benefit on vastus lateralis hypertrophy (at the proximal, middle, and distal sites) for the longer-length training condition. However, in another experiment, after training protocols at longer (0–90° of knee flexion) and shorter (0–50°) average muscle lengths, the authors showed an evident advantage for training at longer muscle lengths only at the distal site of the vastus lateralis [27]. Together, these results indicate that a clear benefit for training at long muscle lengths may occur only when training in that length in an isolated manner, and in comparison to shorter isolated ones [13,16,20,27,29,30]; so that the slight difference produced by the present exercise setups was not sufficient to elicit different adaptations.

The present study has some issues to be addressed. Firstly, the training program included other exercises for the elbow flexors, and this, despite having a high relation to practical settings, might have clouded the true magnitude of the effect of preacher curl training [21]. Moreover, this experiment was carried out in untrained young adults and results cannot be generalized to other populations of different ages, or training statuses, given that responses to training are influenced by such factors [1,24]. Finally, muscle thickness was assessed only at the mid-portion of the biceps, and considering that training-induced hypertrophy may be inhomogeneous [1,17,31], the assessment of more muscle sites (e.g., proximal and distal; short and long heads of the biceps separately) might provide greater insights regarding the hypertrophy responses.

## 5. Conclusions

In conclusion, similar responses to preacher curl training were obtained with CAB and BAR apparatus. The BAR group obtained greater strength gains at 20° of the elbow flexion, while no difference was observed between groups for strength gains at the 60° and 100° positions and the muscle thickness.

The results of this study suggest that coaches and practitioners can choose to perform preacher curl training on a cable-pulley device or with a barbell with the expectation of achieving similar results for strength and hypertrophy. The choice may be based on the availability of equipment or personal preference. If one apparatus is occupied in the weight room, the other can be utilized without diminishing the training effects. Combining both approaches may also be a valid strategy. Moreover, although extrapolations should be done with caution, other similar exercise variations (e.g., lying chest flies on low-pulley cable vs. dumbbells) with which the highest torque is during the final or initial degrees of the movement may induce similar adaptations (to the pectoralis major, for example) as well.

## Supplementary Materials

The following are available online at https://www.mdpi.com/1660-4601/17/16/5859/s1, Figure S1: Overview of the measure of the thickness of biceps brachii muscle.
