# Supplementary figures and images for: Placing Greater Torque at Shorter or Longer Muscle Lengths? Effects of Cable vs. Barbell Preacher Curl Training on Muscular Strength and Hypertrophy in Young Adults
Source: Int J Environ Res Public Health. 2020 Aug 13;17(16):5859. doi: 10.3390/ijerph17165859 (PMC7460162; doi:10.3390/ijerph17165859)

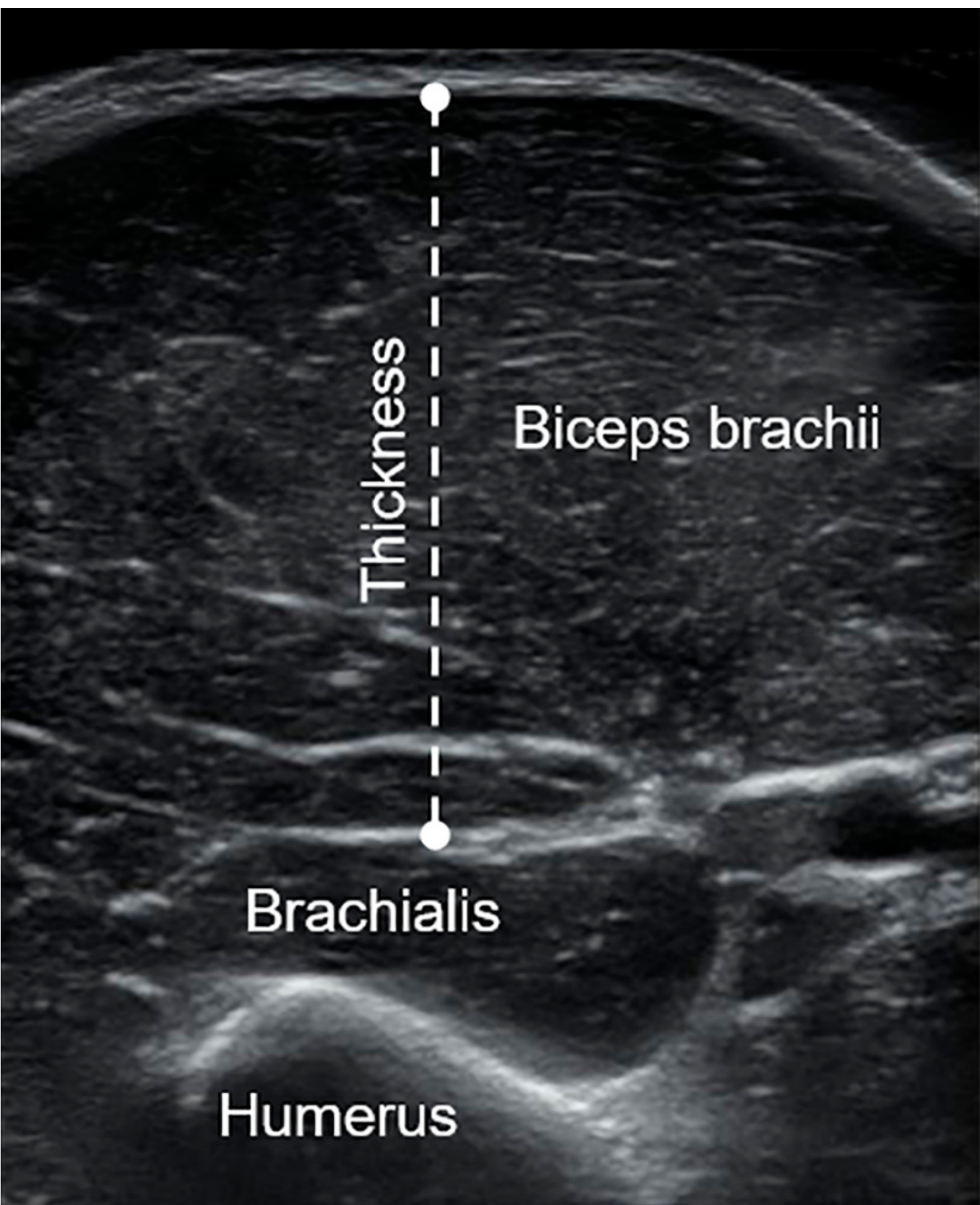

Supplement: Supplementary file 1 [file ijerph-17-05859-s001.pdf]
